# Immune Surveillance by Rhinovirus-Specific Circulating CD4^+^ and CD8^+^ T Lymphocytes

**DOI:** 10.1371/journal.pone.0115271

**Published:** 2015-01-13

**Authors:** John W. Steinke, Lixia Liu, Ronald B. Turner, Thomas J. Braciale, Larry Borish

**Affiliations:** 1 Department of Medicine, University of Virginia Health System, Charlottesville, Virginia, United States of America; 2 Carter Immunology Center, University of Virginia, Charlottesville, Virginia, United States of America; 3 Department of Pediatrics, University of Virginia Health System, Charlottesville, Virginia, United States of America; 4 Department of Pathology, University of Virginia Health System, Charlottesville, Virginia, United States of America; The University of Texas Medical School at Houston, UNITED STATES

## Abstract

**Background:**

It is difficult to experimentally infect volunteers with RV strains to which the subject demonstrates serological immunity. However, in RV challenges, viral clearance begins before *de novo* adaptive immune responses would develop. We speculated that adaptive immunity to RV reflects heterologous immunity by effector memory cells.

**Methods:**

DCs were generated from monocytes using GM-CSF and IL-4 and RV39 loading accomplished with a dose of ∼350 TCID_50_/10^5^ cells. RV-induced maturation was established as modulation of MHC class II, CD80, CD83, and CD86. Circulating RV targeting CD4 and CD8 T cells were investigated as induction of RV-specific proliferation (CFSE-dilution).

**Results:**

Maturation of DC by RV was confirmed as upregulation of MHC Class II (83.3±5.0% to 87.8±4.1%), CD80 (39.4±7.2% to 47.6±7.7%) and CD86 (78.4±4.7% to 84.1±3.4%). Both CD4 and CD8 memory T cells were recognized in the circulation of healthy subjects.

**Conclusions:**

RV drives DC maturation and results in their ability to present RV antigens to both T helper and cytotoxic lymphocytes. Both CD4 and CD8 cells capable of recognizing RV-associated antigens are present in the circulation of healthy subjects where they are presumably involved in immune surveillance and explain the rapid recruitment of an adaptive immune response during RV infection.

## Introduction

In addition to the morbidity associated with being the prime inducer of the “common cold” [1, 2], rhinovirus (RV) infections are the most important cause of asthma exacerbations, especially amongst children and adolescents. In this population, viral respiratory tract infections are associated with 80–90% of emergency room visits for asthma [3–5] with RV accounting for 75–80% [3, 6, 7]. Importantly, up to a third of all RV infections in both non-asthmatics and asthmatics are asymptomatic and, among the asthmatics, asymptomatic infections reportedly rarely lead to exacerbations [8, 9]. Thus, RV infection alone is not sufficient to produce an asthma flare, and most infections, including symptomatic clinically-administered experimental infections, *rarely* lead to exacerbations [3, 6, 8, 10–12]. Multiple explanations have been put forward to explain why only some infections lead to either the development of “cold” symptoms or to asthma exacerbations. One proposal has been that a defect in innate immunity, characterized by the deficient capacity of airway epithelial cells to produce type I and type III interferons (IFNs) as well as interleukin (IL)-15, leads to higher viral load and longer duration of infection [13–16]. Alternatively, RV-associated asthma exacerbations are linked to increased expression of a type 2 cytokine signature (IL-4, -5, and -13) [15, 17] suggesting an alternative (or complementary role) for aberrant adaptive immune responses. These latter studies do not make clear whether this T cell signature is derived from RV-targeting T lymphocytes and, if so, whether these are CD4^+^ T effector (Th2-like) or CD8^+^ cytotoxic (Tc2-like) cells [15, 18]. It must be recognized that it is certainly also plausible that this cytokine signature could have been derived from an RV-mediated expansion of allergen-specific or other bystander T cells or even by innate immune cells, such as eosinophils, basophils, and various natural T cells such as NKT cells and innate lymphoid type 2 (ILC2) cells [19–21].

Understanding the immune mechanisms driving symptomatic RV infections and, similarly, the mechanism of RV-asthma exacerbations is therefore contingent initially upon studying the natural history of infection and the role of innate and adaptive immune responses. We have recently completed studies utilizing a viral challenge model in volunteers to address the natural history of infection including quantification of viral load at regular intervals post infection. These experimental challenges require use of an RV strain to which subjects do not display preexisting immunity by serological testing, as our studies have shown that it is impossible to induce infections in the face of preexisting serotype-specific antibodies [10, 22]. These studies were also striking for the rapidity with which viral titers began to decline post inoculation. As this time frame is too rapid to reflect *de novo* activation of naïve T lymphocytes, we posited that these adaptive immune responses had to represent engagement of effector memory T cells that had developed to a different strain of RV, but which recognized one or more shared T cell epitopes, i.e., heterologous immunity. The current studies were therefore performed to investigate the expression of circulating effector memory T cells engaged in immune surveillance in healthy volunteers.

The primary target of RV infection is respiratory epithelium [23, 24] and RV does not productively infect dendritic cells (DCs) [25, 26]. However, we recognized that following exposure to RV, immature DCs residing in the nasal mucosa would take up RV antigens and subsequently differentiate into mature DCs that would migrate to regional lymph nodes to present viral antigens to pre-existing effector memory RV-specific T lymphocytes. We therefore investigated the hypothesis that even in the absence of productive RV infection, immature DCs can process and present RV antigens and mature into functional antigen-presenting cells. We investigated the ability of RV-differentiated DC to present antigens to both CD4 and CD8 T lymphocytes. And finally, to address whether these RV-specific T cells could drive the cytokine milieu associated with asthma exacerbations, we investigated the expression of intracellular and secreted cytokines consistent with either a Th1 (IFN-γ) or Th2 (IL-4) cytokine “signature.”

## Methods

### Subjects

Heparinized venous blood was obtained from 14 healthy volunteers (18–55 years old). All subjects signed informed consent using protocols approved by the Human Investigation Committee at the University of Virginia for this complete study.

### Monocyte-derived DC maturation and RV stimulation

Peripheral blood mononuclear cells (PBMCs) were isolated through Ficoll-Hypaque (Sigma, St Louis, MO) density centrifugation. CD14^+^ cells were enriched (>95%) from PBMCs using positive magnetic affinity column purification (Miltenyi Biotec, Auburn, CA). Cells were suspended in STEM PRO-34 medium (Invitrogen, Carlsbad, CA) supplemented with GM-CSF (1000 U/ml; BD Pharmingen, San Diego, CA), IL-4 (1000 U/ml; BD Pharmingen), penicillin (10,000 U/ml), streptomycin (10 μg/ml) and 10% autologous serum and maintained at 37° C in 5% CO_2_. Cells were differentiated for 5 days before analysis, with medium and supplements changed at day 3. RV-induced maturation of monocyte-derived DCs was evaluated after the application of ∼350 TCID_50_ of RV39. After an additional 48 hrs, cells were collected for surface expression of maturation markers. In addition, supernatants and mRNA transcripts were isolated and analyzed for expression of cytokines relevant to DC maturation status and anti-viral immunity or involved in T lymphocyte development and immune deviation.

### DC cell surface marker expression

DC cultures were collected and fixed by addition of 2% paraformaldehyde (100 μl) for 10 min followed by staining buffer (5% bovine serum albumin (BSA) and 0.1% sodium azide in PBS). Cells were pre-incubated with mouse IgG (20 μg) to block non-specific binding. Each sample was then labeled with anti-MHC class I-APC, anti-HLA-DR-APC, anti-CD11c-PE, anti-CD14-PE, anti-CD80-PE, anti-CD83-PE, anti-CD86-PE, or anti-CD1c-brilliant violet for 30 min. All antibodies came from BD Biosciences (San Jose, CA) with the exception of anti-CD1c, which was from Biolegend (San Diego, CA). Flow cytometry was performed on a Becton Dickinson six FACS-Canto machine equipped with CellQuest software (BD Biosciences). Intact cells were analyzed based on forward and side scatter. Within this population, viable cells as determined using CellTrace violet (Life Technologies, Grand Island, NY) were used for subsequent analyses. Data were analyzed using FlowJo version 9.4.3 (Tree Star Inc., Ashland, OR) after appropriate gating on the relevant populations.

### Cytokine/growth factor determination

IFN-α2, IL-10, IL-12p40, IL-15, and TNF-α levels in the supernatants of DCs cultured with or without RV were determined using a Millipore bead-suspension assay according to manufacturers instructions (Millipore, Billerica, MA). The sensitivities for these assays were <2 pg/mL. In addition, IFN-γ, IL-4, and IL-13 were measured from the supernatants of activated CD3^+^ T cell-DC co-cultures (described below) by commercial EIA (sensitivities < 0.78 pg/ml; Bio-Rad, Hercules, CA).

### Reverse transcription and quantitative real-time PCR (qPCR) detection of mRNA transcripts

Total RNA was extracted from DCs and qPCR performed as described [27, 28]. Semi-quantitative analyses of changes in gene expression induced by RV were performed using the comparative C_T_ method. Briefly, the amount of target, normalized to an endogenous reference and relative to a calibrator was calculated as 2^ΔΔCT^ with ΔΔC_T_ = (threshold cycle unstimulated gene of interest − threshold cycle unstimulated housekeeping gene) − (threshold cycle stimulated gene of interest − threshold cycle stimulated housekeeping gene). The comparative C_T_ method was validated by confirming that the efficiencies of target and reference amplification were equivalent. Additionally, the data are presented as the ΔC_T_ between the reference gene and target gene of interest. Primers for β-actin, TGF-β1 and IL-10 were as described [27–29]. Primers for IL-1β, IL-12p40 and p35, IL-15, IL-18, TNF-α and TSLP were purchased from Qiagen. Primers for detection of RV infection were as described previously [30]. Briefly, The cDNA was amplified using primers and probes specific for conserved regions of RV and detected by qPCR (RV forward 5′-GGCCCTGAATGTGGCTAA-3′; RV reverse 5′-ATCCCCGCAATTGCTCGTTAC-3′; probe 5′-FAM-CTTGCAGCCAATGCA-BHQ-3′)(Integrated DNA Technologies, Coralville, IA).

### DC-T cell co-culture

CD3^+^ T cells enriched (>99% pure) from PBMCs obtained from volunteers using positive magnetic affinity column purification (Miltenyi Biotec). To determine cell proliferation, carboxyfluorescein succinimidyl ester (CFSE) dye (Invitrogen) was added to the T cells as per manufacturer’s protocol. Co-cultures were set up using 2×10^6^ CD3^+^ T cells/well at 10:1 T lymphocyte:DC ratio using control and RV-loaded DC. Cells were incubated for 7 additional days after which proliferation and intracellular cytokine staining (ICS) was determined. Additional controls included maturing the DCs in the presence of ultraviolet light inactivated (UVi) RV or with poly(deoxyinosinic-deoxycytidylic) acid (125 ng) and using these cells in the T lymphocyte:DC coculture as described above. For the proliferation studies, data were analyzed separately via flow cytometry for CD4^+^ and CD8^+^ T cell populations with data expressed as % CFSE^low^ cells, after subtracting the low background proliferation produced by the autologous mixed lymphocyte reaction (AMLR) observed with the control (unloaded) DC.

### Cell surface and intracellular cytokine staining

For the ICS, T cells were stimulated with phorbol 12-myristate 13-acetate (50 ng/mL) and ionomycin (2 μg/mL; Sigma-Aldrich) for 5 hrs and with Monensin (1 μl/ml; BD Pharmingen) added for the final 4 hrs. Cells were pre-incubated with mouse IgG (20 μg) to block non-specific binding. Each sample was then labeled with anti-CD4-PerCP-Cy5.5 (1:200 μl) and anti-CD8-PE-Cy7 (1:200 μl) for 30 min. Cells were fixed by addition of 2% paraformaldehyde (100 μl) for 10 min followed by staining buffer (5% BSA and 0.1% sodium azide in PBS). ICS was performed with Fix and Perm cell permeabilization kit (Invitrogen) according to the manufacturer’s instructions. Cells were suspended in permeabilization buffer along with mouse IgG (50 μl). For cytokine staining, cells were incubated with either anti-IL-4-PE or anti-IFN-γ-APC-Cy7 (1:100) for 30 min (BD Biosciences, San Diego, CA). The cells were incubated for 30 min and washed with staining solution (5% BSA and 0.1% sodium azide in PBS). Flow cytometry was performed as described with appropriate gating on the CD4^+^ and CD8^+^ populations.

### Statistical analyses

Data were contrasted between control and RV-inoculated samples by independent t-tests with or without equal variances where appropriate using SPSS 20 software (Armonk, NY).

## Results

### RV-Mediated DC Maturation

**Figure 1:**
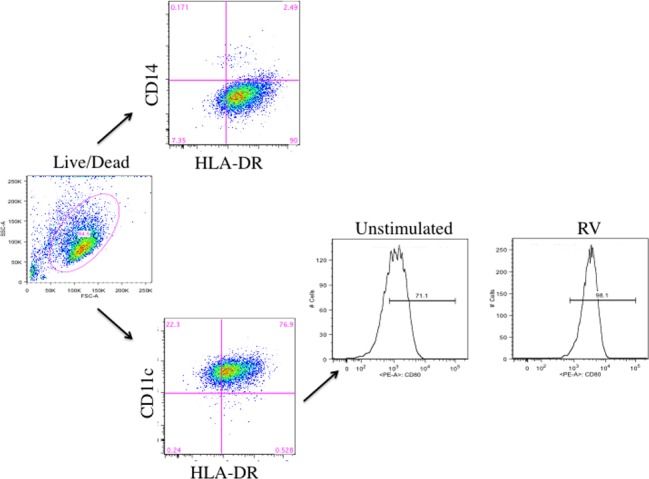
Representative gating strategy used for DCs. After 5 days of culture, monocyte derived DCs were labeled with a dye to mark viable cells. Using flow cytometry, live cells were analyzed for expression of CD14 or CD11c and co-expression of HLA-DR. Cells that were CD14^low^ and CD11c/HLA-DR^high^ were further analyzed for cell surface markers. A representative histogram showing the change in CD80 expression following RV maturation is presented.

**Figure 2:**
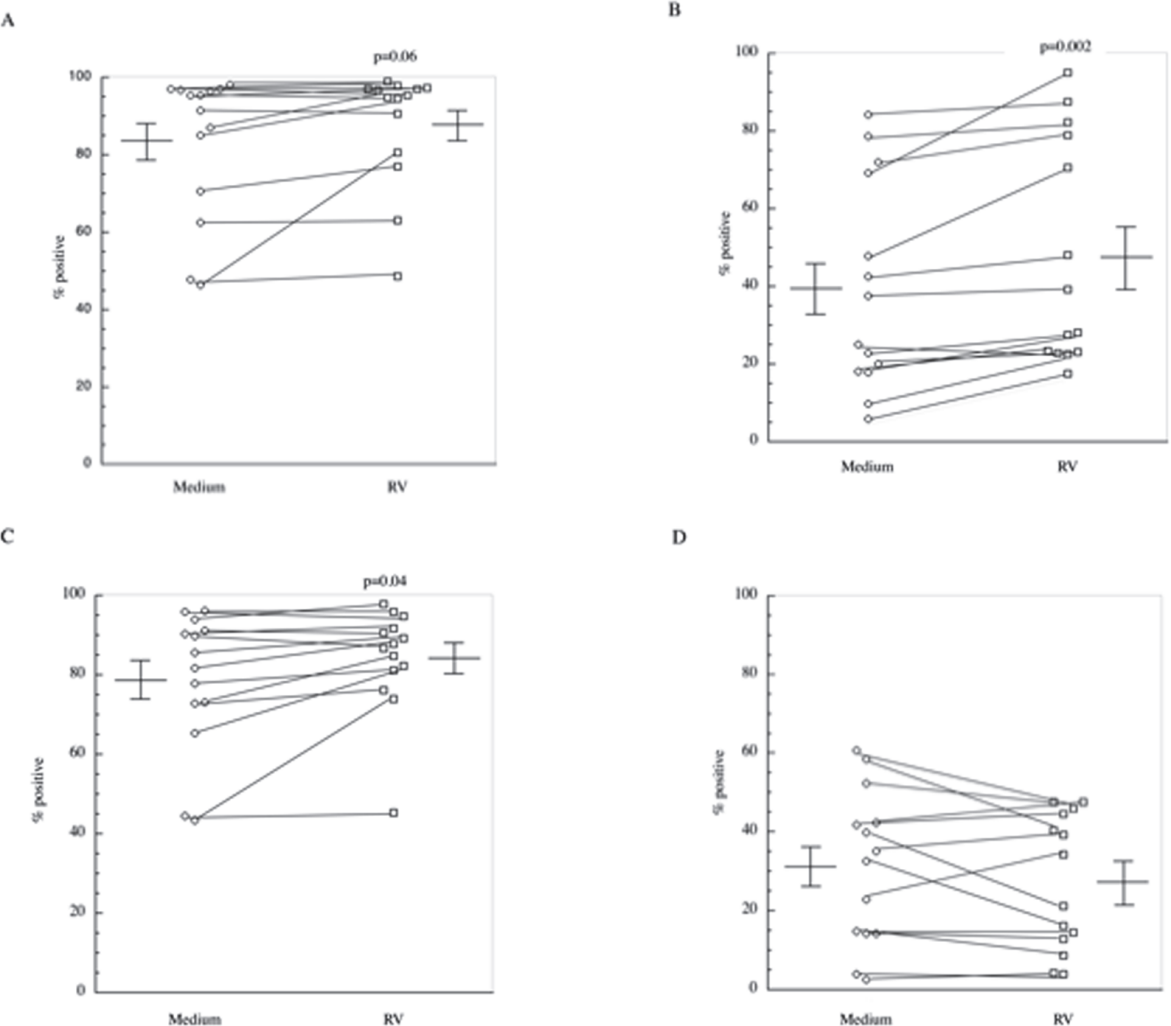
Analysis of DC maturation markers. Cell surface expression of DC activation markers was determined after incubation of cells with ∼350 TCID_50_ (50 μg) of RV for 48 hrs. Data are presented as percent positive for each marker A) MHC Class II, B) CD80, C) CD86 and D) CD83. Individual data points show changes in surface expression from untreated and RV-treated DCs (n = 14).

Monocyte-derived immature dendritic cells were generated from isolated peripheral blood CD14^+^ monocytes by culturing the cells with IL-4 and GM-CSF for 5 days. A representation of our gating strategy for DCs is shown in Fig. 1. A dye was used to mark cells and only live cells were analyzed. For analysis of cell surface markers, cells that were CD11c^hi^ and HLA-DR^hi^ were used. Cells harvested from these cultures displayed a characteristic cell surface phenotype of dendritic cells as compared to input CD14 cells i.e., MHC class I^hi^, CD11c^hi^, CD14^absent/low^ and CD1c^+^ (not shown) and displayed cell surface expression of MHC class II, CD80, CD83, and CD86 (Fig. 2). Consistent with previous reports [26], we confirmed that DCs are not infected by RV, as we failed to detect any amplification of the RV genome or expression of negative sense complementary RNA (by qPCR) in DC exposed to increasing inoculum doses of purified infectious RV (not shown). To assure antigen availability and optimal maturation of the DC, we incubated the cells with a large inoculum i.e., ∼350 TCID_50_ (50 μg) of enriched RV/10^5^ DC for 48 hrs at 37°. Following this stimulation regimen, MHC Class II expression by the DC was increased in 11/14 subjects (83.3±5.0% to 87.8±4.1%; p = 0.06), CD80 expression was increased in 13/14 subjects (39.4±7.2% to 47.6±7.7% p<0.02) and CD86 expression was increased in 10/14 subjects (from 78.6±4.7% to 84.1±3.5%; p<0.04)(Fig. 2A-C). In contrast, although not achieving statistical significance, CD83 expression decreased from 31.0±5.2% to 27.2±4.6% (Fig. 2D). Incubation of DCs in the presence of UViRV also increased maturation markers to the same extent as RV itself (data not shown). Incubation of DCs with RV or UViRV did not alter cell viability as the percentage of live cells was unchanged or slightly increased (medium 77.8±9.3%; RV 82.4±6.8%; UViRV 83.7±5.9% (n = 3)).

### DC Cytokine Transcript Expression

Changes in DC gene expression associated with innate immunity and immune deviation were measured (Table 1) and RV-pulsed DCs exhibited significant increases in IL-15, IL-12p35, IL-10, and TGF-β1 mRNA compared to control untreated DC cultures.

**Table 1:** Change in Cytokine Gene Expression in RV-Pulsed DC.

Cytokine	Δ CT Medium	Δ CT RV	Fold change
Innate			
TNF-α	4.76±0.67	4.82±0.91	0.96
IL-1β	7.67±0.51	7.23±0.58	1.36
IL-15	7.77±0.47	6.04±0.90	3.32*
Th1 promoting			
IL-12p35	8.88±0.95	7.83±0.97	2.07*
IL-12p40	6.63±0.72	6.86±0.70	0.85
IL-18	4.43±0.67	4.60±0.74	0.89
Th2 promoting			
TSLP	9.59±1.21	10.59±1.5	0.5
Immune suppressive			
IL-10	7.04±0.64	6.56±0.50	1.39*
TGF-ß1	3.47±0.49	2.44±0.63	2.04*

*p<0.05

### DC Cytokine Secretion

When cytokine secretion was examined following incubation of DC with RV, modest increases in IL-15 (3.0±0.4 to 3.7±1.0) and IFN-α2 (9.2±6.2 to 22.0±16.0) were observed but these did not achieve statistical significance.

### DC-T Lymphocyte Co-Culture

**Figure 3:**
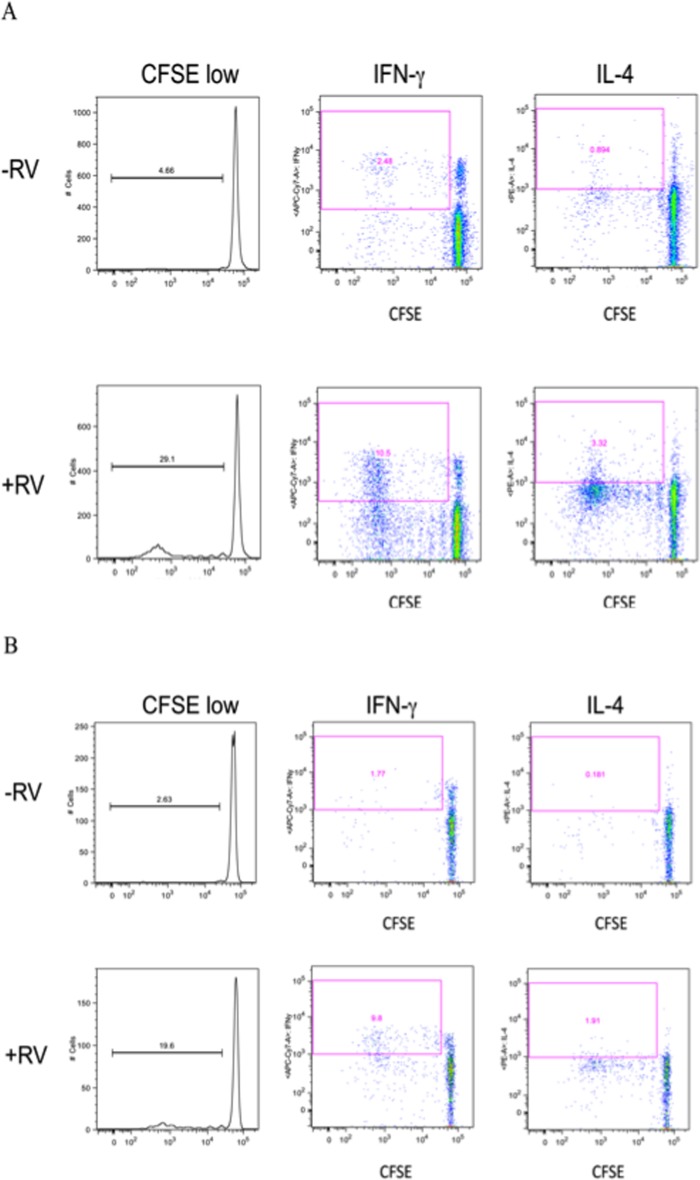
RV-specific T cell proliferation in healthy volunteer. Representative flow cytometry showing IL-4 and IFN-**γ** expression in T cells (CD4 (2A) and CD8 (2B)) that were co-cultured with RV-pulsed DCs for 7 days. The x-axis displays cell proliferation as measured by CFSE dilution with the y-axis demonstrating intracellular cytokine expression.

**Figure 4:**
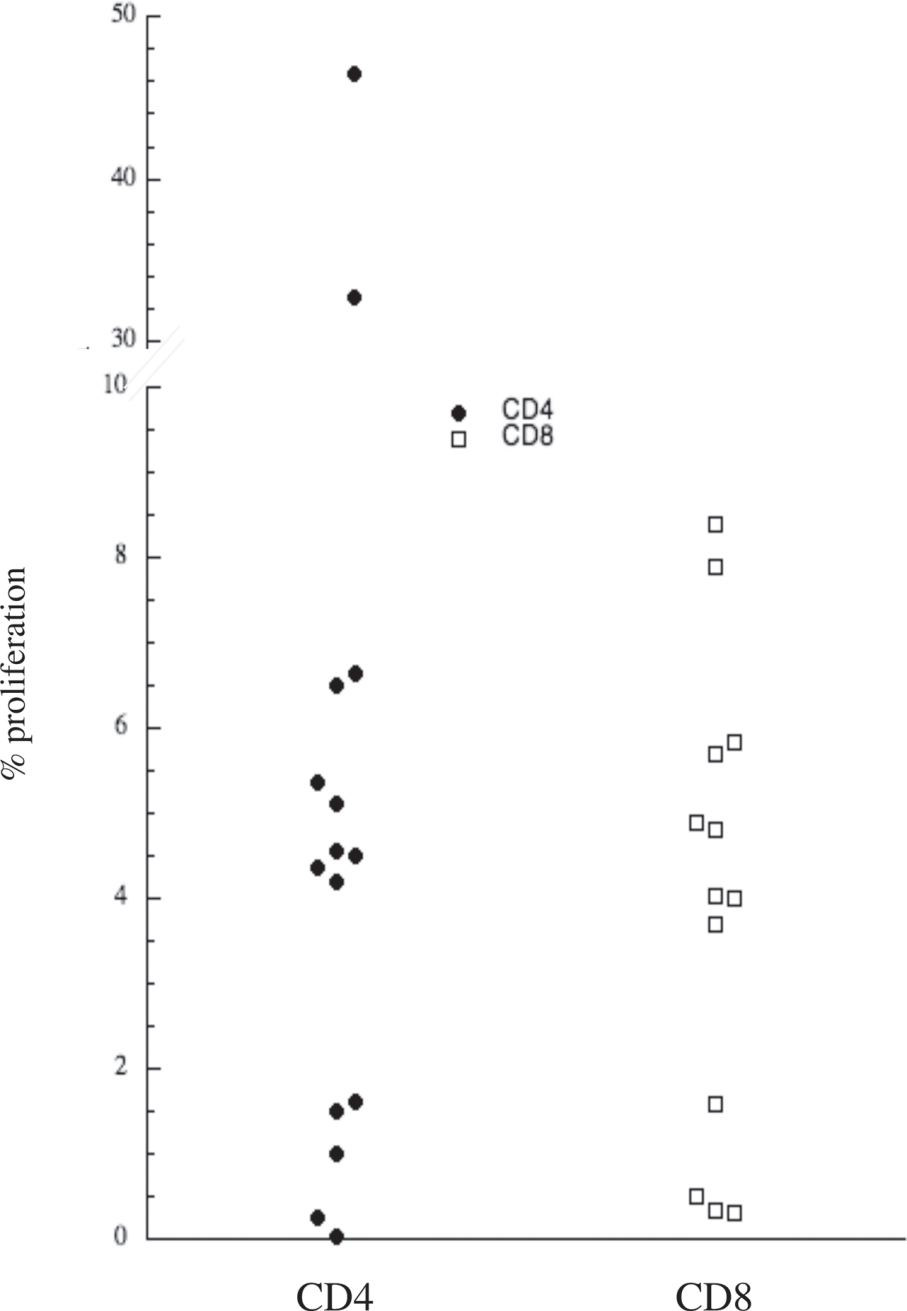
Percent of CFSE^low^ CD4^+^ and CD8^+^ RV-specific T lymphocytes in healthy volunteers. CFSE-labeled CD3^+^ lymphocytes and RV-loaded DC were co-cultured as described for 7 days. Data display percent of CFSE^low^ T lymphocytes separately gated to analyze CD4^+^ and CD8^+^ populations after subtracting background proliferation induced by unloaded DC.

Having established that DCs could be matured *in vitro* by exposure to RV, we utilized these matured DCs to investigate the expression of circulating RV-specific T lymphocytes in the circulation of healthy subjects. RV-loaded DCs were used to activate CFSE-labeled CD3^+^ T lymphocytes. T lymphocyte-DC co-cultures were carried out for 7 days after which proliferation was determined as CFSE dilution and cytokine expression examined in CD4^+^ and CD8^+^ populations via ICS. Representative flow cytometry data displaying proliferation and cytokine expression (IFN-γ and IL-4) for CD4^+^ and CD8^+^ T cells are displayed in Fig. 3. Low levels of T cell proliferation were observed with control (RV^−^ DC), presumably reflecting an autologous mixed lymphocyte response (AMLR). However, 14/15 healthy volunteers demonstrated variable degrees of RV-specific cellular proliferation amongst their CD4^+^ lymphocytes and 15/15 in their CD8^+^ cells (Fig. 4 and Table 2). In addition, both IFN-γ as well as lesser amounts of IL-4 intracellular cytokine expression were identified within the proliferating cell populations following stimulation with RV. When secreted cytokines were quantified via EIA this was significantly greater for IFN-γ, IL-4, and IL-13 (Table 3) following RV infection. These results were specific to active RV as DCs matured with ultraviolet light inactivated RV or with poly(deoxyinosinic-deoxycytidylic) acid did not induce T cell proliferation or intracellular (IL-4 or IFN-γ) cytokine expression (not shown).

**Table 2:** Proliferation and Cytokine Expression in Immune Surveillance.

	**CD4**	**CD8**
CFSE low (%)	8.34±3.4	3.46±0.74
IFN-γ^+^ (% of proliferating cells)	74 ± 9	45 ± 10
IL-4^+^ (% of proliferating cells)	39 ± 8	6 ± 2

**Table 3:** Concentration (pg/ml) of Cytokines in Control and RV-Loaded DC-CD3^+^ T Lymphocyte Co-Cultures.

	Control	RV^+^
IFN-γ	101.1±33.1	1232.0±386.4*
IL-4	5.6±1.2	19.0±5.4*
IL-13	50.5±17.1	147.0±33.2^†^

*p<0.05

^†^p<0.01

## Discussion

Rhinovirus infection is responsible for most asthma exacerbations in children and adolescents [3, 6, 7], however, surprisingly little is known regarding the immune and inflammatory mechanisms underlying these exacerbations. One proposed mechanism is that a defect in innate immunity in asthmatics leads to more severe infections [13–16]. In part to address the role of such a deficient innate immune response, in our recently completed studies we quantified viral load in the nasal secretions of inoculated volunteers [30]. These studies were striking for the demonstration that RV titer consistently peaked at ∼4 days post infection, presumably reflecting the initiation of adaptive immune responses. As demonstrated by serological testing, none of these patients had recently been infected with the RV serotype used for these challenges (RV39) insofar as we recognize that it is not possible to induce infections in volunteers having serotype-specific antibodies [10, 22]. Similarly, *de novo* immune responses by newly activated naïve T cells do not develop prior to day 7 post infection. We therefore posited that these adaptive immune responses were mediated by effector memory cells generated in response to a structurally related RV strain that shared an epitope with RV39 that, as such, would be capable of driving heterologous immunity. The current studies were performed to investigate expression of circulating RV-specific T effector lymphocytes in healthy subjects, capable of mediating immune surveillance. To recapitulate naturally occurring infections, we utilized RV to mature DCs. Previous reports [25, 26] confirmed here, demonstrate that DCs are not infected by RV. However, this does not preclude the ability of the virus to induce DC maturation including through engagement of molecular pattern receptors for viral RNA (TLR3, TLR7, RIG-I, MDA5, and likely others) and also to process RV antigens through both MHC Class I and MHC Class II presentation pathways. These studies demonstrated that exposure to the RV (and UViRV) did mature the DC as evinced by increased expression of MHC class II, CD80, and CD86 (Fig. 2). In contrast, although not achieving statistical significance, CD83 levels were decreased. While often shown to increase with non-virally-mediated DC activation, CD83 tends to remain unchanged or even decrease with viral infections, including RV [26, 31, 32]. DC maturation was further evinced as changes in expression of cytokine mRNA (Table 1). This included upregulation of IL-15, a cytokine central to anti-viral immunity through its role in orchestrating recruitment and activation of both NK cells and CD8^+^ cytotoxic T cells [16]. Similarly, induction of IL-12 would likely contribute to the more prominent engagement of Th1-like lymphocytes. The expression of more immune suppressive cytokines (IL-10 and TGF-β) may appear paradoxical, but it is now recognized that co-expression of these cytokines in an anti-viral immune response may be critically important in ameliorating bystander tissue damage [33]. Modulation of expression of these transcripts was not confirmed as secreted proteins, but this may reflect the challenges of accurately assessing concentrations of secreted proteins accumulating in supernatants, including their capture by surface receptors and secreted binding proteins or protease degradation and the timing of secretion in relation to mRNA changes.

Most importantly, we demonstrated the functional capacity of RV-matured DC through their capacity to induce T cell proliferation. For these studies, we were also eager to investigate the activity of both CD4^+^ T helper and CD8^+^ T cytotoxic lymphocytes. As the prime instigators of anti-viral immunity, it is likely that CD8^+^ T cells would have a central role in an adaptive anti-RV immune response. However, due to the challenges of utilizing autologous APCs that utilize MHC class I processing pathways, the expression of RV-specific CD8^+^ T cells has never been addressed in humans, a problem circumvented by our use of autologous monocyte-derived DCs. As shown in Figs. 3 and 4, these studies variously induced T cell proliferation amongst both the CD4^+^ and CD8^+^ lymphocytes. While variability is the norm in human research, one explanation for the very high levels of circulating CD4^+^ RV-specific cells observed in a few subjects (Fig. 4) is that even though all our subjects reported being asymptomatic and not having recent “cold” symptoms, it remains possible that they did, in fact, have a concurrent or recent RV infection. This is consistent with our experience that up to half of all RV infections are minimally or asymptomatic. Our results differ slightly from those of Kirchberger et al. who saw inhibition of T cell responses when RV was used to prime DC-T cell cocultures [25]. This is likely due to numerous factors including differing RV strain, low RV levels used to prime the DCs, and the high background of spontaneous T cell proliferation in the absence of antigen.

The presence of both CD4^+^ and CD8^+^ cells in the circulation of our healthy subjects capable of recognizing and proliferating in response to RV39 is consistent with the rapid (day 4 post infection) engagement of an adaptive immune response after experimental inoculation with this virus strain and as such, the engagement of a heterologous immune response.

In addition to defective innate immunity, RV-induced asthma exacerbations are associated with expression of a cytokine signature associated with Th2-like lymphocytes [15, 17]. In our studies, experimental RV infection in asthmatics induces a rapid increase in total IgE and an influx of eosinophils, consistent with the biological activities of IL-4, IL-5, and IL-13 [10 and unpublished observations]. These studies did not address whether these cytokines are being produced by RV-specific lymphocytes (or indeed, if these are even being produced by lymphocytes at all, as opposed to eosinophils, mast cells, NK T cells, ILC2 cells, or other cell types). Furthermore, if produced by T effector cells, these could certainly be RV-specific lymphocytes, although it is certainly plausible that this could reflect activation of bystander cells, e.g., allergen-specific T cells. For example, RV upregulates IgE (FcεRIα) receptor expression on DCs and thereby could promote IgE-facilitated antigen uptake and presentation to allergen-specific T cells [34]. However, we were eager to address the expression by RV-specific T cells themselves of cytokines associated with both Th1 and Th2 cytokine signatures in both the CD4^+^ and CD8^+^ cohorts. We speculated that as an important cell driving viral eradication, RV-specific cytotoxic cells could be a prime source of “Th2” cytokines. Such cells, termed Tc2-like lymphocytes, mediate eosinophilic inflammation during anti-viral immune responses [35–38]. Their expression could be an additional mechanism of RV-mediated asthma exacerbations through their propensity to concomitantly be a source of type 2 cytokines while mediating their anti-viral responses. These studies do demonstrate both intracellular and secreted cytokine expression of primarily IFN-γ (Fig. 3, Tables 2 and 3), consistent with the type I immune-deviating profile of the DC. Intriguingly, however, various degrees of IL-4, IL-5, and IL-13 intracellular and/or secreted proteins were observed in both the CD4^+^ (Th2-like) and CD8^+^ (Tc2-like) T lymphocyte populations (Fig. 3, Tables 2 and 3). These studies were performed in non-allergic, non-asthmatic volunteers. By utilizing this strategy in asthmatics it should be possible to interrogate their T cell response and to define the distinct features of the RV-specific T cells in these individuals. It may then be feasible to determine whether asthmatics – in particular those who go on to develop an experimental RV-induced asthma exacerbation – are even more prone to express such a Th2 and/or Tc2 profile.

As noted above, UViRV resulted in DCs that matured to the same extent as RV-loaded DCs, so the inability of the DCs to activate a T cell response is not due to defects in expression of maturation molecules. This may not be surprising as the UV irradiation of RV would preserve their expression of pathogen-associated molecular patterns and thereby continue to drive DC maturation and activation. The absence of a T cell response is therefore likely due to a defect in the ability of these matured DCs to activate the T cells. Several possibilities could explain this. While RV does not replicate in the DCs, translation of the RV genome may occur and may be necessary for optimal peptide loading onto MHC molecules. With UViRV, this translation is less likely to occur. Alternatively, the UVi process crosslinks viral capsid proteins and alters the way they are processed, again likely leading to inefficient loading of the MHC molecules. Either way the end result is lack of T cell activation.

In summary, while not productively infecting DCs, we demonstrated the ability of RV to drive DC maturation and their ability to present RV antigens to both CD4^+^ T helper and CD8^+^ T cytotoxic lymphocytes. Both CD4 and CD8 cells capable of recognizing RV-associated antigens are present in the circulation of healthy subjects where they are presumably involved in immune surveillance and thereby can explain the rapid recruitment of an adaptive immune response after RV infection.
